# Process evaluation of the systematic medical appraisal, referral and treatment (SMART) mental health project in rural India

**DOI:** 10.1186/s12888-017-1525-6

**Published:** 2017-12-04

**Authors:** Abha Tewari, Sudha Kallakuri, Siddhardha Devarapalli, Vivekanand Jha, Anushka Patel, Pallab K. Maulik

**Affiliations:** 1 The George Institute for Global Health, 311-312 Elegance Tower, Jasola, New Delhi, 110025 India; 2 The George Institute for Global Health, University of Oxford, Oxford, UK; 3 The George Institute for Global Health, Sydney, Australia; 4 University of New South Wales, Sydney, Australia

**Keywords:** Common mental disorders, Mental health services, mHealth based interventions, Low and middle income countries, India, Process evaluation

## Abstract

**Background:**

Availability of basic mental health services is limited in rural areas of India. Health system and individual level factors such as lack of mental health professionals and infrastructure, poor awareness about mental health, stigma related to help seeking, are responsible for poor awareness and use of mental health services. We implemented a mental health services delivery model that leveraged technology and task sharing to facilitate identification and treatment of common mental disorders (CMDs) such as stress, depression, anxiety and suicide risk in rural areas of the state of Andhra Pradesh, India. The intervention was delivered by lay village health workers (Accredited Social Health Activists – ASHAs) and primary care doctors. An anti-stigma campaign was implemented prior to this activity. This paper reports the process evaluation of the intervention using mixed methods.

**Methods:**

A mixed methods pre-post evaluation assessed the intervention using quantitative service usage analytics from the server, and qualitative interviews with different stakeholders. Barriers and facilitators in implementing the intervention were identified.

**Results:**

Health service use increased significantly at post-intervention, ASHAs could followup 78.6% of those who had screened positive, and 78.6% of the 1243 Interactive Voice Response System calls made, were successful. Most respondents were aware of the intervention. They indicated that knowledge received through the intervention empowered them to approach ASHAs and share their mental health symptoms. ASHAs and doctors opined that EDSS was useful and easy to use. Medical camps organized in villages to increase access to the doctor were received positively by all. However, some aspects or facilitators of the intervention need to be improved, including network connectivity, booster training, anti-stigma campaigns, quality of mental health services provided by doctors, provision of psychotropic medications at primary health centers and frequency of health camps.

**Conclusion:**

The respondents’ views helped to understand the barriers and facilitators for improving the likely effectiveness of the intervention using Andersen’s Modified Behavioral Model of Health Services Use, and identify the mechanisms by which those factors affected mental health services uptake in the community.

**Trial registration:**

The study is registered with Clinical Trials Registry India (Applied - 16/07/14-Ref2014/07/007256; registration received - 04/10/17-CTRI/2017/10/009992).

**Electronic supplementary material:**

The online version of this article (10.1186/s12888-017-1525-6) contains supplementary material, which is available to authorized users.

## Background

Globally, mental disorders account for 8.5% of the total Years of Life Lost due to premature death and Years Lived with Disability [1]. It is estimated that 75–85% people with mental disorders in low- and middle-income countries (LMIC) do not receive effective treatment – the ‘treatment gap’ - and mental health is often the lowest health priority in those settings [2]. Even though effective treatment exists for mental disorders, lack of trained mental health professionals, poor infrastructure, ineffective government policies, low awareness and increased stigma related to mental health are likely to be important contributors to this treatment gap [3]. This is worse in rural settings [4]. Prior research has shown that task shifting and use of electronic decision support systems (EDSS) can enable mental health services delivery [5–7]. Using similar principles, we conducted a project that focused on a mental health services delivery model to screen, diagnose and manage common mental disorders (CMDs) such as stress, depression, anxiety and suicide risk - the Systematic Medical Appraisal, Referral and Treatment (SMART) Mental Health Project [8].

SMART Mental Health Project was conducted across two rural sites, a Scheduled Tribe (ST) Area and a non-Scheduled Tribe Area, of Andhra Pradesh, a south-Indian state [8, 9]. Scheduled Tribe communities are identified by the administration based on their unique cultural and linguistic characteristics, which deem them to be an indigenous community, hence guaranteeing special administrative rights and status. Andhra Pradesh has about 6 million ST population, and this group has poorer health indicators such as life expectancies, under-5 mortality, and many others, compared to other rural communities in the area [10].

Overall, the project had two key objectives: 1) The development of a multifaceted intervention using training, task shifting, and mobile-based decision support to increase the screening, treatment and referral of individuals with CMDs, and 2) Evaluation of the intervention after implementation in 30 ST villages, to provide preliminary evidence of effectiveness, feasibility, acceptability and potential for scale up. The SMART Mental Health study findings indicated an improvement in awareness about mental health in the community; increased screening of common mental disorders by ASHAs; increased use of mental health care provided by primary health care doctors (increased from 0.8% at baseline to 12.6% at post-intervention); and reduction in depression and anxiety scores at post-intervention for those who were screened positive for depression and/or anxiety, at baseline [9].

This paper reports on the mixed methods process evaluation for the SMART Mental Health Project.

## Methods

The purpose of the process evaluation was 1) to describe the experiences of those exposed to the intervention and 2) to identify the barriers and facilitators in implementation of the intervention.

### Project plan

**Fig. 1:**
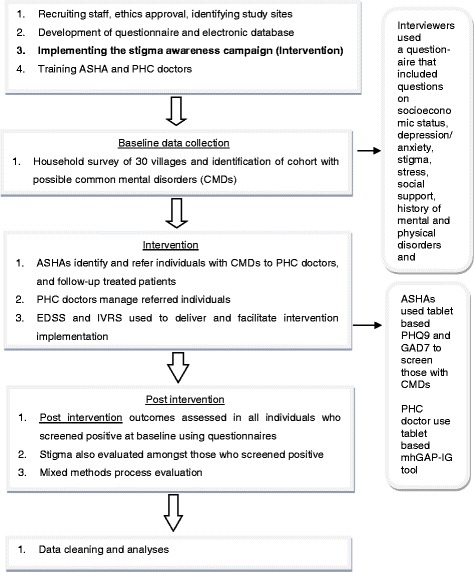
Overall project plan

The project was divided into different stages as outlined in Fig. 1. The intervention had a number of facets as outlined below and shown in Fig. 1.

1. Mobile technology based EDSS: Two separate EDSS were developed for screening by ASHAs, and clinical diagnosis and management by primary care doctors. The tool used by ASHAs was based on the Patient Health Questionnaire (PHQ9) [11] and Generalized Anxiety Disorder questionnaire (GAD7) [12]. The diagnosis and management guidelines used by the doctors was based on the Mental Health GAP – Intervention Guide (mhGAP-IG) [13]. Both tools were developed as applications on a 7-in. Android tablet using an Open MRS platform. Both the PHQ9 and GAD7 provide diagnoses of mild/ moderate/ severe levels of depression/anxiety based on cut-off scores [14]. Only scores ≥10 on either scale, or a positive response to the question on self-harm in the PHQ9, were considered as screen positive for this project. Patients were either seen at primary health centers (PHCs) or at the health camps in the villages. Any patient with severe mental disorders or any other complications were referred to the district hospital which had trained mental health professionals. Data captured on the EDSS were shared securely between the ASHAs and doctors, and it allowed the ASHAs to monitor the progress of the cases referred by them using through a traffic light system that helped them prioritized the patients.
2. Interactive voice response system (IVRS): An algorithm based IVRS sent out pre-recorded messages to the screen positive individuals to visit the PHC doctor to seek care or continue treatment as advised by the ASHA or the doctor. Messages to the ASHAs and doctors reminded them to screen and follow up individuals as per guidelines.
3. Stigma Reduction Campaign: A campaign was conducted prior to the baseline survey to increase mental health knowledge and reduce stigma in the community for 8 weeks across all villages. It included a number of strategies: sharing brochures and posters on mental health with the community using a door-to-door campaign; showing a video of a person talking about his own mental illness and a video of a film actor talking about CMD; staging live performances of a drama on mental disorders and the help seeking in 2 villages, while showing video recordings in the other villages. Discussions followed each presentation.

### Total project duration and eligibility criteria

The total project duration was for 24 months from May 2014 to April 2016 including a formative phase where the interventions were tested and refined [15]. The project was conducted in 30 villages associated with two PHCs, located in an ST area of the West Godavari district of Andhra Pradesh [9]. Fifteen villages from each PHC were randomly selected and then it was ensured that each village had adequate number of ASHAs proportionate to the population. One village with a population > 3000, was replaced by another one with a smaller population, as the village did not have sufficient number of ASHAs to cater to the population. All eligible adults ≥18 years of age who gave informed consented to participate and were not suffering from any severe physical disorder that limited them from accessing mental health services, were included.

### Process evaluation

The process evaluation was undertaken from January to February 2016. Quantitative data on service usage from the backend data stored on servers, was acquired throughout the intervention to understand frequency and type of services used - type and appropriateness of care provided by doctors, frequency of follow-up of screen positive cases by ASHAs, and numbers and success rate of IVRS calls. Qualitative data was collected using focus group discussions (FGDs) and in-depth interviews (IDIs) of key stakeholders. Consolidated criteria for reporting qualitative research (COREQ) guidelines for reporting qualitative research was followed [16]. A complete checklist has been uploaded as additional file (Additional file 1: Table S1).

The study participants in the process evaluation consisted of community members, ASHAs, primary care doctors, village leaders and field staff. We included all doctors and field staff in our interviews. However, community members and village leaders were selected purposively. The selection of participants ensured representations of all villages and PHCs, and we tried to ensure equal participation of both genders. We contacted all screen positive adults who had received the intervention through either PHC and divided them by gender. Since it was harvesting season, not all adults were available but we ensured that information was sent out to most. Only those community members, ASHAs, and village leaders who were available and willing to participate were included in the interviews. FGDs and IDIs were conducted either at community centers in the villages, or in the house of the interviewee.

The FGDs and IDIs guidelines comprised of a set of questions developed by the research team through discussions and were translated to Telugu (Additional file 1: Table S2). The questions were relevant to the different processes of the study and probes helped clarify some of the points. The participants were assured about the confidentiality of their responses. A trained moderator conducted the interviews after introducing the process to the participants. A note taker was responsible for audiotaping the discussion and taking notes. Each FGD and IDI lasted for about 35–40 min. Each interview was audio taped, and later transcribed and translated into English to ensure the reliability of data. No individual identifiers were stored or analysed.

### Ethics approval and consent to participate

Ethics approval was received from the Independent Review Committee of the Centre for Chronic Disease Control, New Delhi, India. Informed written consent was obtained from all participants. Data were collected according to the Declaration of Helsinki on ethical research. Approval for conducting the project was obtained from the Health Department, Government of Andhra Pradesh, and the Integrated Tribal Development Agency was informed about the project. Approval was also obtained from all the local village administrative bodies.

### Data analysis

**Fig. 2:**
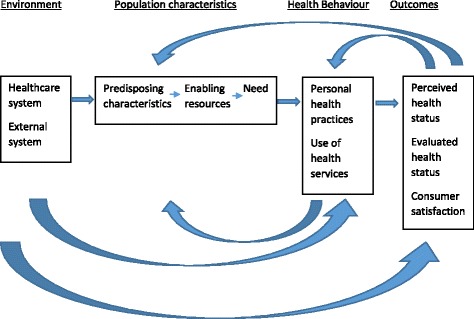
Andersen’s modified behavioural model of health services use

Quantitative usage specific data was downloaded from the server, cleaned, and analyzed using descriptive methods. Several strategies were used to ensure that the qualitative data analyses were systematic and verifiable. Initially, the interview guide was used to identify some thematic areas which were refined subsequently as analyses progressed. Two researchers (SK and AT) coded transcripts independently and the themes identified by each of them were discussed in detail, compared, and refined, to generate the themes and sub themes based on consensus. This ensured the inter-rater reliability and reduced bias. Thematic analysis based on grounded theory was used to analyze data [17, 18]. *NVIVO* was used to analyse the data [19]. Barriers and facilitators were plotted onto Andersen’s Modified Behavioral Model of Health Services Use [20], which posits a set of factors – environment, population characteristics, health behavior and outcomes, which interact in a dynamic manner to determine health services use (Fig. 2).

## Results

ASHAs were able to follow up 78.6% of all screen positive cases in the 3 month period. Altogether 1243 calls were placed to the community, ASHAs and doctors, of which 78.6% were successful (heard by them). Of these 1243 calls, 1043 were made to the community (success rate of 71.3%) and 200 were made to ASHAs and doctors (success rate of 43%). Doctors appropriately managed all cases as per the algorithm and only referred cases with moderate depression requiring antidepressants to the district hospital, due to lack of medications at the primary health center [9].

The qualitative evaluation included seven FGDs and six IDIs. Four FGDs were conducted with community members, separately for males and females (males - *n* = 14, aged 34–62 years; females - *n* = 23, aged 28–65 years). Two FGDs were conducted with ASHAs (female-*n* = 16, aged 29–62 years), and one FGD with the project field staff who monitored the activities of the ASHAs and doctors, which included both sexes (*n* = 19, aged 19–49 years). Two IDIs were conducted with the two PHC doctors and four IDIs with the community leaders of four villages (Male and female - aged 29–53 years). The results from both FGDs and IDIs are described below according to identified themes and grouped under facilitators, barriers, and further suggestions related to the overall implementation process.

### Facilitators

#### Knowledge of the project and CMD

Most community members were aware of the different activities of the project, suggesting that the project was helpful, and that they learnt new things related to causes and treatment of mental illness. Community members shared that prior to this project they were not aware where to go and whom to approach for CMDs. Some community members highlighted that this project helped people to discuss and share their problems with others, which in turn reduced anxiety and stress in those individuals.

*“We have got a lot of awareness about this project. We did not know what a mental illness is until they [project staff] came and told [us]. When they [project staff] came we explained to them about our physical pains, etc. and during a medical camp [a] doctor saw me and informed me about the treatment. (Community member, FGD5)*

*“This project was excellent. We learnt all about the causes of mental illnesses and what kind of treatments should be taken for such [mental disorder] problems”. (Community member, FGD-4) “If we discuss our problems with someone, it make us happy and we feel good”. (Community Member-FGD7)*

ASHAs added that the increased knowledge and awareness about CMDs empowered the community members to approach ASHAs and share their mental health problems. Doctors indicated that the project was a learning experience for them as they gained knowledge about different treatments for CMDs.

*“We have learnt many things from this project. Initially we did not know the importance of mental disorders [CMD] and we had been treating people for other disease conditions, and through this study we got an idea about dealing and treating people with common mental disorders” (Doctor, IDI 4).*

### Benefits of involving ASHA’s

Almost all the community members and doctors were aware of the role of ASHAs in the project. The majority of the community members shared that involving an ASHA was helpful as she was known to the community, making it easier for them to share their problems. This was considered to not have been possible with the involvement of any unknown person or outsider. Some community members appreciated that the ASHAs went to each house for screening.

Community members shared that ASHAs not only paid repeated visits to enquire about their health, but also tried to motivate them to visit the doctor. They believed that ASHAs were sensitive and courteous in asking questions. One community member shared,

*“ASHAs know everything well. ASHAs are the right people as they keep moving around the village and are familiar with the village problems” (Community member, FGD2)*

Both doctors involved in the intervention believed that ASHAs rapport with the community helped them to play a major role in implementing the intervention. They believed that this rapport helped them to motivate individuals to seek treatment. They felt that ASHAs accompanying patients to the PHC and briefing the doctor about the patient’s condition was especially helpful.

“*ASHAs are not new to us and our relationship with them [ASHAs] have been good. So they [ASHAs] used to give a lot of information about the patient, [at times] more than the patients. Patients [at times] did not discuss properly. As ASHAs are locals, they know what is happening in the families of the patients [and so could provide a context]”. (Doctor, IDI2)*

“*Yes … ASHAs did a good job, they worked hard in motivating the patients. They used to accompany patients to the PHC and they used to help me understand the person’s condition. So that was very helpful”. (Doctor, IDI5)*

### Impact of training

The majority of participants including ASHAs, doctors and field staff reported that, overall, the training provided was useful. Training sessions helped them to operate the tablets and record observations on a regular basis. ASHAs elaborated that the training helped them to do their job and increased their confidence in approaching people to share their mental health problems. It also made them aware about mental disorders in general and CMDs specifically. They indicated that training on conducting interviews and operating the tablets was useful, even though they were initially apprehensive about participating because of their education levels.

*“We have never done any interviews before so we got to know how to do it and how to approach someone and explain about the study…about mental disorders” (ASHAs, FGD1)*

Doctors indicated that the training facilitated easy use of the mhGAP-IG tool.

*“This training programme was very detailed and we learnt about the treatment pattern and new tool which was easy to use” … “Because of this training programme, now I know what kind of treatment is to be given to what cases and which cases have to be sent to the specialist which are beyond my ability”*. (*Doctor, IDI5*)

The field staff mentioned that training helped ASHAs to improve their communication skills which in turn helped ASHAs persuade community members to participate in the intervention. Some field staff opined that most ASHAs who were unable to navigate a tablet initially, were able to collect data using the tablets easily as a result of the training.

*“Most of them did not even know how to operate a mobile phone, and because of this training it helped them to operate the tablets and click on the various options” (Field staff, FGD4)*

### Organizing medical camps in community

Most community members and ASHAs appreciated organizing medical camps in villages. They felt that these camps helped people to understand their health status and facilitated access to doctors within their own village. Some community members believed that these camps not only provided health services but created an environment where the entire community were sensitized about issues related to mental disorders. A few ASHAs mentioned that in these camps, when people with similar problems interacted, they shared their problems with each other and discussed ways to overcome their problems. Village leaders felt that the camps created awareness about the importance of seeking treatment and visiting the doctor.

*“Because of these medical camps we [community members] get treatment here, rather than going somewhere. So it is easier for us”. (Community member, FGD5)*

*“If such medical camps are conducted, people will get to know about their health condition, and will know at what stage they are, and where to seek information for getting treatment for their condition”. (Village leaders, IDI3)*

### Use of technology - EDSS /IVRS

Using the EDSS and receiving IVR messages were appreciated by the community members, doctors and ASHAs. The majority of ASHAs and doctors opined that EDSS was useful and was easy to use. Initially, there were some difficulties in understanding certain terminology, but these were clarified by trainers. Doctors shared that the duration of assessing a patient was initially 30–40 min due to the length of the questionnaire, but with practice could be completed within 15–20 min. The doctors believed the technology helped them to diagnose patients with CMD.

*“The overall use of the app was very comfortable and now I cannot see any problems from the app perspective, overall it was a good experience”. (ASHAs-FGD4)*

*“Yes, definitely it was a great learning experience for me. I have used this tool for patients who came to me to get treated for some mental disorders”. (Doctor, IDI 5)*

Some community members and ASHAs believed that IVRS messages were useful for patients. These messages reminded patients to visit the PHC or to follow other instructions given by doctors and ASHAs.

*“Using IVR to send the messages to patients is good as it will be useful to remind people to visit the PHC or to follow instructions”. (Community members, FGD4)*

### Barriers

#### Stigma & discrimination to receive treatment

Some participants talked about the challenges faced by the community to receive treatment. Community leaders indicated that people were not fully aware of mental disorders, hence were not interested in revealing their mental health problems. They felt that those with mental health problems and even their families were often subject to stigma and discrimination which is a barrier to seeking treatment. For example, difficulties in securing matrimonial alliances for their children may be an issue if someone is diagnosed with mental disorder in the family. Some community members shared that there were some who were hesitant to visit a doctor, or disclose their problems even after visiting the doctor, because of getting “*labeled as a mad person”.* A few community members felt that mental illness cannot be cured by going to a doctor.

Field staff felt that the villagers did not reveal their problems completely in spite of assurance about confidentiality. They reported that community members did not visit the PHC due to stigma, and for the same reason they either did not reveal their mental health symptoms to the doctor or only mentioned their physical symptoms when visiting the PHC.

ASHAs shared that they had experienced some negative reactions from the community members while asking questions and had to spend significant time convincing people to respond. They also shared that although privacy was ensured, community members feared that their family members or neighbours might overhear or might come to know about their illness, which may complicate their social problems.

*“Many people did not ‘open up’ completely. Even though we assured them [community people] about keeping all the information confidential, so I felt we should create more awareness so that they understand the problem better and come out with their problems in a better way”.* (*Field staff, FGD 4)*

#### Financial livelihood and social constraints

Doctors viewed that poor economic conditions and lack of financial stability were major reasons preventing greater benefits from the intervention. Village leaders expressed that people did not go to PHCs due to financial reasons and lack of transport. They elaborated that most of the community members are daily wage earners and hence are unable to go to the PHC without financial loss.

*“We would need facilities near our places [to avoid] bearing the costs of the travel expenses, and medicines.”(Community members, FGD3).*

Some members mentioned that people go to PHC for physical illnesses but ignore mental illnesses. Other community members mentioned that patients did not visit the PHC as they did not trust the doctors’ abilities to treat them.

ASHAs expressed that they faced difficulty in finding people for screening as often the villagers went to the fields for agricultural work in the morning until the evening, hence making it challenging to find people during the day for interviews.

*“Yes, [madam] most of us had faced the issue…where we were not able to find people for the doing the interviews. We used to start doing the interviews at 5 [o’clock] in the evening…as nobody was available during the day time…..most of them used to go for agricultural work” (ASHA’s, FGD2)*

The other problem that the ASHA faced was related to faulty social perceptions because of the caste system. According to them, higher caste community members believed that mental illness occurred among ‘laborers’ who were generally from low caste communities, and was not an issue in their ‘high caste communities’, hence they were reluctant to answer the questions. Some ASHAs also shared that they faced difficulty in posing the question on suicide as most community members responded poorly to being asked such questions. ASHAs further added that problems exist but people did not express them freely as they were apprehensive about where to seek treatment and what whether they would have to leave their work to get treated.

#### Gaps in using technology–based applications/IVR messages

Majority of the participants including ASHAs, community members, field staff and village leaders shared that most of the households had one mobile phone in the family which is often left at home when going out to the fields, or is with another family member, hence is often not available to the patient to receive calls or IVR messages. Other reasons cited by the community members, ASHAs and field staff for not receiving or responding to IVRS were - calls from unidentified numbers, fear of losing talk time due to connecting to those calls, lack of network connectivity, and lack of awareness about handling the mobile phones.

*“Usually there are some calls from the mobile companies; if we receive the calls then we were asked to click on some option; because of which some service get activated and our balance (money) get deducted for activation of those services”. (Community members, FGD 5)*

Doctors shared that sometimes they found mhGAP-IG tool difficult as they were unclear how to complete options based on the patient’s response. They also added that there were some difficulties in understanding certain terminology using the app.

Field staff felt that poor network was one of the major problems in this area, as most villages were remote and lacked good connectivity.

*“We are unable to use mobile phones because of network and signal [connectivity] problem. Another issue is even if we get such calls we do not pay so much of attention as we are not aware of such things”. (Community members, FGD6)*

### Suggestions to improve the project

Almost all participants suggested the need to create more awareness on mental health issues including help seeking. Most considered medical camps as an appropriate strategy to create awareness among community members. They suggested a need to conduct more medical camps in each village so that it can be accessed by larger number of people.

*“If it [camps] is organized in each panchayat [local administrative body] it will be good. If there is a need to go to the PHC they will not go as it is very far, so if there is a camp in [a] village nearby, definitely people will come for treatment”. (Village leader, IDI1)*

Some community members also recommended that the intervention should be continued for a longer period of time with support from government. Some community members said that they should be informed about the IVRS call number in advance prior to its implementation. Prior intimation of camps through IVR messages was also recommended. A few community members felt that there should be more involvement of village leaders so that they can motivate people to participate in such projects.

*The project should be done by taking support from other organizations and the village leaders [and] administration, which will help in the success of the project. (Village leaders, IDI3)*

Another suggestion was that when the patients visit the PHC, appropriate treatment should be provided. This would help in building the trust of the community in the health system.

Nearly all ASHAs and doctors suggested a need to conduct booster training every six months.

## Discussion

The process evaluation showed that the interventions delivered as part of the project were feasible and acceptable. A number of facilitators and barriers were identified. To our knowledge, this is the first study from an ST area reporting on the process evaluation of an intervention on mental health services delivery. We reached thematic saturation, and respondents identified facilitators and barriers of the intervention that complements the quantitative data presented earlier [9]. Implementing this intervention in a specifically remote area as this, while may limit generalizability across India due to its unique logistical and geographical challenges, provides valuable insight into how to implement similar health delivery models in similar remote settings for other health conditions.

The barriers and facilitators can be framed using Andersen’s Modified Behavioral Model of Health Services Use [20] (Table 1). While the components - environmental, population characteristics, and health behavior - affect outcomes, the outcomes themselves provide a feedback loop that affects perceived need and health behavior.

**Table 1:** Identifying barriers and facilitators of mental health services use based on Anderson’s Modified Behavioral Model of Health Services Use [20]

Key component from Andersen’s model	Scenario prior to the project	Intervention and processes implemented	Respondents perception about the intervention as mentioned in the process evaluation – positive (+)/ negative (−)
*Environmental*			
Healthcare system	• PHCs were not providing any mental health care • ASHAs did not have any knowledge about CMDs • Primary care doctors lacked adequate knowledge and skills to identify and manage CMDs • Patients needed to travel to PHCs to get treated, leading to increased expenses and loss due to time spent in travel and waiting	• 21 ASHAs and 2 doctors were trained on using the mobile technology based applications • Training and supervision provided to both ASHAs and doctors to use the applications • An algorithm based EDSS implemented to facilitate screening by ASHAs • The mhGAP-IG based EDSS facilitated the doctors ability to manage CMDs • Health camps organized in villages to facilitate easier access to doctors • An algorithm based followup system developed for ASHAs to ensure treatment adherence	• Community members and village leaders felt that project was helpful (+) • Community members were able to share their mental health symptoms with ASHAs (+) • The ASHAs felt empowered by their enhanced skills acquired through training (+) • The doctors increased their knowledge and expertise to manage CMDs (+) • Additional booster training was suggested by ASHAs and doctors to supplement the current training and help them identify issues for improvement; current one time training was suggested as being g less than optimal (−) • Majority of participants appreciated the role of ASHAs and doctors (+) • The ASHAs repeatedly followed up with patients and enquired about their health which was appreciated by the community (+) • Health camps were appreciated as they reduced time and money spent in going to the PHCs (+)
*Population characteristics*			
Predisposing characteristics	• Poor knowledge about CMD in the community • Most community members worked in the fields during the day which prevented data collection by field staff or screening by ASHAs or help seeking if needed	• A mental health awareness campaign organized using multimedia processes • Personalized and dramatized narratives of mental illness used along with traditional posters and brochures and video of a local film actor talk about the project • Both field staff and ASHAs often interviewed community members late in the evening after they returned from work • Community members had to migrate in search of jobs	• The community members mentioned that prior to the campaign they were neither aware of CMDs nor knew where to seek treatment (+) • Community members, community leaders, ASHAs, doctors and field staff confirmed that the mental health awareness program was useful (+) • However, some community people were not interested in revealing their health problems completely due to stigma (−) • Inspite of using evenings to contact community members who were in the field due to their work, at times others could not be contacted even after repeated attempts as they had migrated out of the villages (−) • A belief persisted amongst some community members that CMDs were a problem amongst lower socio-economic status (−) • Some community members had reservations about the doctors ability to provide adequate treatment (−)
Enabling resources	• No pre-existing mental health services in the village • Community were not oriented towards identifying CMDs • No treatment was sought from PHC for any psychological problems • Getting treated at PHCs was both time consuming and involved travel expenses	• Village leaders and local administration were kept informed about the project at each step • Local health staff – ASHAs and doctors used to provide care, and no additional resources were recruited for treatment purpose • Field staff trained using standard operational procedures and their activities monitored regularly • Field staff monitored ASHAs regularly and ensured the quality of data collected by them; supervisors followed up with doctors regularly to check for any problems that they might be facing with the application • Health camps in villages enabled patients with CMD to seek care from doctors closer to home • Supervisor coordinated with the doctor and ASHA about the health camps	• Village leaders appreciated the project (+) • Using ASHAs and doctors in primary care for providing the intervention were seen as a positive move by most community members including ASHAs and doctors (+) • ASHAs were found to be particularly useful because – they made repeated visits; used their knowledge about the community while explaining the case to the doctor; accompanied the patient to the doctor (+) • All respondents supported health camps (+) • Health camps were also seen as a place where patients discussed problems amongst themselves and sought peer-led advice on an informal basis (+) • The quality of training and its value for field staff, ASHAs and doctors were underlined by them (+) • Quantitative data showed that a large number of population were screened (>5000), there was significant increase in the proportion of screen positive individuals seeking treatment from doctors (+)
Need	• Perceived need to seek care for CMD was negligible as awareness about CMD was absent • Health workers including PHC doctors were not trained to identify or manage CMDs • No mechanism to increase the perceived need of those with CMD	• Mental health awareness activities and screening of the whole population by ASHAs led to increase in help seeking • The ability of primary health workers including doctors to identify and manage CMD was enhanced by using evidence-based algorithm driven EDSS • 1243 IVRS calls were attempted to remind screen positive individuals about treatment adherence and ASHAS and doctors about regular followups	• With increased perceived and evaluative need, identification of CMD and uptake of services was increased (+) • The treatment provided by doctors and provision of such through health camps also helped to increase ability of the community to seek care (+) • ASHAs provided brief suggestions to cope with stressful situations (+) • The EDSS was found to be acceptable and easy to use by ASHAs and doctors (+) • The mhGAP-IG based doctors app was found a bit time consuming by doctors at least initially (−) • IVRS was opined as a positive move to enhance care (+) • Only 65% of attempted IVRS calls were successful due to various reasons: i. Some community members failed to receive calls as they were either apprehensive about the source of the call or assumed that it will cost them in form of loss of talk time (−) ii. Mobile phones were at times not with the screen positive person as someone else had them, as only one phone was shared in the household (−) iii. Network connectivity was patchy across the villages leading to call drop (−)
*Health Behaviour*			
Personal health practises	• Stigma related to mental health and help seeking • Poor knowledge about CMDs amongst community members and health workers	• A campaign to increase mental health awareness and reduce stigma organized • Enquiring about suicide was a sensitive issue during the intervention	• Overall the campaign was beneficial (+) • Everyone opined that the project led to increased awareness about CMDs and the need to seek care, and led to more people visiting doctors (+) • Some community members did not seek treatment because they continued to be apprehensive about the kind of treatment they would receive (−), or stigma associated with help-seeking (−) • Many community members found the suicide question to be negative and did not like to respond (−)
Use of health services	• No treatment for CMDs in PHCs	• The intervention had a focus on increasing mental health services use for CMDs • Task shifting was used to enable mental health care for the rural population • Technology driven platforms were used to facilitate provision of mental health services • A system developed to ensure followup by ASHAs and doctors • Only 3 camps could be organized in the short time period	• Overall the interventions were thought to be useful by all (+) • Reluctance to seek care to avoid being marked as a family with mental disorders thus jeopardizing the ability to get their children married off (−) • ASHAs and doctors worked collaboratively to provide care (+) • ASHAs were deemed as instrumental to the intervention by everyone (+) • The EDSS and IVRS were seen as facilitating the intervention (+) • Medical camps facilitated increased service use (+)
*Outcomes*			
Perceived health status	• Community members were unaware about CMDs	• A comprehensive mental health intervention implemented	• Most respondent felt that the intervention led to greater perception about CMDs in the community (+) • Some community members were not convinced about seeking care or being screened even after the mental health awareness campaign (−)
Evaluated health status	• No screening or treatment provided at primary care level for CMDs	• All components of the intervention had a primary care level focus	• Mental health services use was increased significantly; depression and anxiety scores reduced significantly (+)
Consumer satisfaction	• No measure of consumer satisfaction in the community	• A pre-post evaluation of the project provided objective assessment of the outcomes	• Most respondents felt that the intervention was beneficial in not only providing increased awareness about CMDs but also the need for seeking care (+) • Some community members highlighted that the project helped then to discuss and share their problems with others which in turn helped those individuals (+) • The role played by ASHAs and doctors were seen positively (+) • Repeated followup by ASHAs was appreciated by the community as a process that motivated the community to access care (+) • Organizing medical camps in villages was appreciated (+)

### Environmental factors related to healthcare system

The training provided to ASHAs and doctors about CMDs was a key facilitator for the project. Using primary healthcare staff to provide mental health services was appreciated by the community and village leaders. Task shifting of the activity - identification and treatment of CMDs - to primary healthcare workers by enhancing their capacity supports the principles of the National Mental Health Programme (http://mohfw.nic.in/sites/default/files/9903463892NMHP%20detail_0.pdf), National Mental Health Policy [21] and World Health Organization’s Mental Health Action Plan 2013–2020 [22]. An earlier review had also indicated some positive effects of task-shifting in mental health [5]. The perceived positive effect of mobile-technology enabled EDSS supports similar findings from earlier research [6, 7]. However, it was apparent that booster training was needed for ASHAs and doctors on a periodic basis, to improve performance. Even though, only 3 medical camps could be organized due to the short period of the intervention, preliminary data show that 19 out of the 30 patients who visited the doctor, did so at the camps, which underlines the value of camps. The qualitative interviews also showed that besides helping in reducing travel time and associated expenses, the camps were also a platform that increased awareness and helped those attending to share their experiences informally amongst themselves and learn coping strategies from peers.

### Population characteristics (predisposing characteristics, enabling resources, need)

The *whole population including health staff* had poor awareness about mental health and CMDs prior to the intervention. The anti-stigma campaign and the steps taken to increase mental health awareness benefited the population, as was evident in changes in knowledge, attitude and behaviours and stigma perception related to help-seeking [9]. This was also observed in the evaluation of the campaign in another similar population within the same district [23]. However, some individuals believed that mental disorders mainly affect the lower socioeconomic status. This incorrect perception may have been a barrier to collecting information about CMDs from them.

Some key *enabling factors* were the involvement of ASHAS, organizing medical camps, and support of local administration. Organizing medical camps in the villages helped those seeking care, as they did not have to travel and lose out on daily wages, and it also saved time. Both expenses and extended travel time to seek care from health facilities are key barriers in rural and remote areas, and given the remoteness of the ST area and poor public transport facilities in the area, the value of the medical camps was greater in this community.

The *perceived need* for seeking care for CMD was enhanced by increasing awareness and the capacity of health workers to screen CMDs and treat them. Use of mobile applications and IVRS facilitated that process even further as it empowered the health workers and primary care doctors by providing them an evidence based tool, and the IVRS messages facilitated the process of follow-up. However, the lack of good quality network connectivity and non-availability of mobile phones with everyone in the family were barriers in implementing the IVRS successfully. One way to improve connectivity is by mapping hotspots in the community using online applications. Increasing the proportion of successful IVRS calls by community members who do not have access to a phone at all times, could be achieved by identifying specific time slots in the day when such individuals are more likely to have phone access. It was also clear that the IVRS number should be shared upfront so that everyone is aware of the source of the call.

### Health behaviours including personal health practices and use of health services

The mental health awareness campaign and the extra effort put in by the ASHAs to allow people to respond comfortably to the screening questions helped to reduce the stigma related to mental health. ‘Suicide’ was especially viewed as being too negative and discussing it as not being culturally appropriate. However, the ASHAs were specifically trained in being sensitive while asking the ‘suicide’ related question. While talking about death is considered as a taboo in many cultures, in India, the additional burden of stigma related to mental health, and the legal implications of suicide (which was until recently an illegal activity liable for punishment) may have been additional factors that prevented people from discussing about such issues freely. More information needs to be shared with the community about the importance of psychosocial management. It may be that since medicines were not prescribed commonly, it was thought that ‘treatment was not appropriate’.

While generally the community were supportive of the role of ASHAs and doctors, some individuals were still sceptical about the skills of the doctors to manage CMDs, though the doctors themselves felt that the training enhanced their skills to manage CMDs. It was apparent that stigma was still a major factor that prevented people from accessing care, even though the need for help-seeking was present.

### Outcomes related to perceived and evaluated health status and consumer satisfaction

Most community members and health workers felt that the intervention had a positive impact on the community. This was further evident in that there was increased service use and reduced scores on depression and anxiety scales following the intervention [9].

## Conclusion

Process evaluations are essential in assessing the implementation of a system-level intervention. IVRS and medical camps appear to be appropriate and favoured strategies to mobilise communities and positively motivate screen positive individuals to avail mental health services. Mobile technology based applications were also deemed as beneficial, though there were some initial problems in navigating the system. Booster training for all staff and health workers, regular medical camps and better strategies to make IVRS messages more effective were identified as key enhancements needed prior to implementing the next steps to maximise the likelihood of effectiveness of the intervention. While awareness about the different intervention components including the anti-stigma campaign was present, more sustained effort is needed to reduce stigma related barriers in the community. Differential accessibility to mobile phones amongst family members is another issue that needs to be addressed by identifying strategies to overcome that barrier. Poor network connectivity should also be addressed when implementing the study in larger areas.

## Additional file

Additional file 1:
**Table S1.** Consolidated criteria for reporting qualitative studies (COREQ): 32-item checklist. The Table outlines the COREQ Criteria that were fulfilled in this study. **Table S2.** Focus Group discussions (FGDs) and in-depth Interview (IDIs) guidelines. This Table lists the questions used in all the qualitative interviews. (DOCX 32 kb)

## Abbreviations

ASHA: Accredited Social Health Activist
CMD: common mental disorder
COREQ: Consolidated criteria for reporting qualitative research
EDSS: electronic decision support systems
FGD: focus group discussion
GAD7: Generalized Anxiety Disorder – 7 item
IDI: in-depth interview
IVRS: Interactive voice response system
LMIC: in low- and middle-income countries
mhGAP-IG: Mental Health GAP – Intervention Guide
PHC: primary health center
PHQ9: Patient Health Questionnaire – 9 item
SMART: Systematic Medical Appraisal, Referral and Treatment
ST: Scheduled Tribe
