# Supplementary material for: Process evaluation of the systematic medical appraisal, referral and treatment (SMART) mental health project in rural India
Source: BMC Psychiatry. 2017 Dec 4;17:385. doi: 10.1186/s12888-017-1525-6 (PMC5715622; doi:10.1186/s12888-017-1525-6)
Supplement: Additional file 1: — Table S1. Consolidated criteria for reporting qualitative studies (COREQ): 32-item checklist. The Table outlines the COREQ Criteria that were fulfilled in this study. Table S2. Focus Group discussions (FGDs) and in-depth Interview (IDIs) guidelines. This Table lists the questions used in all the qualitative interviews. (DOCX 32 kb) [file 12888_2017_1525_MOESM1_ESM.docx]

**Table S1: Consolidated criteria for reporting qualitative studies (COREQ): 32-item checklist**

Developed from: Tong A, Sainsbury P, Craig J. Consolidated criteria for reporting qualitative research (COREQ):a 32-item checklist for interviews and focus groups. International Journal for Quality in Health Care.2007. Volume19, Number 6:pp.349-357

| No | Item | Guide questions/description | Response |
| --- | --- | --- | --- |
| **Domain 1: Research team and reﬂexivity** | | | |
|  | *Personal Characteristics* |  |  |
|  | Interviewer/facilitator | Which author/s conducted the interview or focus group? | SK and SD conducted the interviews and FGDs |
|  | Credentials | What were the researcher’s credentials? E.g. PhD, MD | MSc and PhD |
|  | Occupation | What was their occupation at the time of the study? | Research Fellow & Research Assistant –George Institute for Global Health- India |
|  | Gender | Was the researcher male or female? | Male and Female |
|  | Experience and training | What experience or training did the researcher have? | Facilitators had prior experience of conducting the Interviews and Focus Groups. One of the author (AT) is a Social scientist with experience in qualitative research provided in-person training in conduction of Interviews and Focus Groups and analysis to SK and SD. |
|  | *Relationship with participants* |  |  |
|  | Relationship established | Was a relationship established prior to study commencement? | A prior relationship between the facilitator and participants did not exist in most cases. |
|  | Participant knowledge of the interviewer | What did the participants know about the researcher? e.g. personal goals, reasons for doing the research | Prior to any data collection, we provided all potential participants with an overview of the SMART Mental Health study prior to signing of the informed consent. |
|  | Interviewer characteristics | What characteristics were reported about the interviewer/facilitator? e.g. Bias, assumptions, reasons and interests in the research topic | The moderators introduced themselves at the beginning of each interview and FGD by name, occupation and educational status. |
| **Domain 2: study design** | | | |
|  | *Theoretical framework* |  |  |
|  | Methodological orientation and Theory | What methodological orientation was stated to underpin the study? e.g. grounded theory, discourse analysis, ethnography, phenomenology, content analysis | We used Grounded Theory to inductively derive the themes. |
|  | *Participant selection* |  |  |
|  | Sampling | How were participants selected? e.g. purposive, convenience, consecutive, snowball | Purposive sampling |
|  | Method of approach | How were participants approached? e.g. face-to-face, telephone, mail, email | Face –to -face |
|  | Sample size | How many participants were in the study? | A total of 41 community members, 16 ASHAs, 19 field staff and 2 doctors participated in the study |
|  | Non-participation | How many people refused to participate or dropped out? Reasons? | None |
|  | *Setting* |  |  |
|  | Setting of data collection | Where was the data collected? e.g. home, clinic, workplace | In the community |
|  | Presence of non-participants | Was anyone else present besides the participants and researchers? | Our protocol did not allow non participants. Any non-participants were immediately requested to leave if they entered the room where an interview or focus group took place. |
|  | Description of sample | What are the important characteristics of the sample? e.g. demographic data, date | Demographic data of the participants as they represented the diverse group |
|  | *Data collection* |  |  |
|  | Interview guide | Were questions, prompts, guides provided by the authors? Was it pilot tested? | Yes |
|  | Repeat interviews | Were repeat interviews carried out? If yes, how many? | No |
|  | Audio/visual recording | Did the research use audio or visual recording to collect the data? | Digital audio recordings were made and transcribed verbatim |
|  | Field notes | Were ﬁeld notes made during and/or after the interview or focus group? | Field notes were made during the interview and FGD by the note taker. |
|  | Duration | What was the duration of the interviews or focus group? | Each FGD and IDI lasted for about 35-40 minutes |
|  | Data saturation | Was data saturation discussed? | Yes |
|  | Transcripts returned | Were transcripts returned to participants for comment and/or correction? | No |
| **Domain 3: analysis and ﬁndings** | | | |
|  | *Data analysis* |  |  |
|  | Number of data coders | How many data coders coded the data? | Two coders (SD & AT) coded the data |
|  | Description of the coding tree | Did authors provide a description of the coding tree? | We have done the non-hierarchical coding and the description is provided in text |
|  | Derivation of themes | Were themes identiﬁed in advance or derived from the data? | Themes were derived from the data |
|  | Software | What software, if applicable, was used to manage the data? | NVivo 9 was used for data management and analysis. |
|  | Participant checking | Did participants provide feedback on the ﬁndings? | No |
|  | *Reporting* |  |  |
|  | Quotations presented | Were participant quotations presented to illustrate the themes / ﬁndings? Was each quotation identiﬁed? e.g. participant number | Yes |
|  | Data and ﬁndings consistent | Was there consistency between the data presented and the ﬁndings? | Yes, we have attempted to present our findings in this work clearly in a manner that was consistent with the data collected. |
|  | Clarity of major themes | Were major themes clearly presented in the ﬁndings? | Yes, the findings are presented in the result section. |
|  | Clarity of minor themes | Is there a description of diverse cases or discussion of minor themes? | NA |

**Table S2: Focus Group discussions (FGDs) and in-depth Interview (IDIs) guidelines**

| **FGD with Community members** |
| --- |
| 1. Give us your opinion about the Smart Mental Health Programme conducted by George Institute, Hyderabad in your community.  - *Can you recall any of the activities (Probe: ASHAs visit, screening, IVR messages and medical camps).* - *Did you attend /participated in any of the activities*  1. Did you share the information with your family, friends, colleagues or neighbors? If yes, what was their opinion? If no, what were the reasons?   **Screening & Camps**   1. What was your experience when ASHAs visited you for asking the questions/ screening? Please give details .*Probe:*  - *Were you comfortable in responding those questions? Please state the reasons.* - *Was it useful? If yes, how? If no, please elaborate what was not useful?* - *Do you think ASHAs are the best person to ask these questions? If yes, why? If no, please suggest who will be the best person to ask these questions and why?*  1. Did you visit the doctor of your PHC or any? If yes, what was your experience?  - *Was it useful? If yes, how? If no, please elaborate what was not useful?* - *What can be improved?*  1. What was the support you got from the ASHAs and George Institute staff to visit the PHC?  - *Was it useful or not?? If yes, how? If no, please elaborate what was not useful?* - *What can be improved?*  1. What was the support you got from the ASHAs and George Institute staff for follow up? Please specify the reasons? *Probe:*  - *Was it useful or not? If yes, how? If no, please elaborate what was not useful?* - *What were the ways that could be changed?*  1. In your opinion what are the reasons for follow up patients visiting or not visiting the PHC for continuing treatment? 2. Are you aware of medical camps organized by George Institute in your community??If yes, Please share your experience?  - *Were these camps easy to access?* - *Were doctors easy to approach and consult?* - *Would you like to receive such services in future also? If yes, why*   **App/IVR**   1. Have you received any IVR message as part of project activity? If yes, how was your experience in receiving the IVR messages? *Probe:*  - *Was the information useful? If yes, in what ways?* - *Did you find any difficulty in understanding them? Probe : language and content of the message* - *Would you like to receive similar messages in future? If yes, why?? If no, please give reasons?* - *If you have not received this please share the reason for not receiving it?*   **Suggestions**   1. Do you think this programme should be continued, modified, or discontinued? State your reasons. 2. Please give your suggestions to improve the programme in future? |
| **FGD with ASHAs** |
| **Trainings**   1. Please tell us about your experience with Smart Mental Health Programme conducted by George Institute, Hyderabad. 2. How far was the training helpful in carrying out activities? *Probe:*  - *What was helpful in the training?* - *What were the aspects and issues that you felt needed more discussion?* - *How does this training help in improving your skills?* - *Do you have any suggestions for future training programme?*  1. In your opinion what are the various factors that encouraged and discouraged individuals in seeking treatment?  *Probe: age groups, gender and socio, economic status*   **App/IVR**   1. How was your experience in using the app/tablet provided by the George as part of project activity? *Probe:*  - *Was training helpful in handling apps and tabs, likes and discomforts?* - *What level of support did you receive from GI staff to handle app/tablet and how?* - *What were the positive and negatives?* - *How do we improve this support?*  1. Did you receive IVR messages? If yes, how was your experience in receiving the IVR messages*? Probe*  - *What were the positive and negative experiences?* - *How do we improve this?* - *If you have not received this please share the reason for not receiving it.*   **Implementation**   1. Please tell us about the difficulties that were faced while screening the individuals? 2. Could you please share your experience in motivating the screen positive to visit the doctor? 3. What challenges did you face in following up with those who were screen positive 4. Overall do you think this way of screening and follow up was useful or not? *Probe:What were the ways that could be changed?* 5. Do you think this intervention must be continued, modified, or discontinued? State your reasons  - *Has this been useful to increase your skills?* - *Would you be checking for CMD using basic questions like “how one is feeling” in future? Probe: If yes, how? If no, why?*   **Suggestions**   1. Please give your suggestions how can we improve the programme in future? |
| **FGD with field investigators & project staff** |
| 1. Please tell us about your experience with Smart Mental Health Programme conducted by George Institute Hyderabad. *Probe*  - *Role in programme,* - *interaction with project staff, ASHAs, and community members and Doctors*   **Training**   1. How was your experience in conducting training for ASHAs conducted by George Institute? *Probe:*  - *How useful or not useful was it?* - *How it can be improved?* - Any other aspects and issues that you felt needed more discussion? - *Any suggestions for future training programme?*   **Implementation**   1. In your opinion what are the reasons for follow up patients visiting or not visiting the PHC for continuing treatment? 2. *What was the level of support you received from GI staff, ASHAs , community members and Doctors ? Probe*  - *Was this support useful* - *How can it be improved*  1. *What was your experience in working with ASHAs? Please share your positive as well as negative experience. Probe:*  - *Interaction with people, handling the tablet, motivate people to visit doctor,Any other*  1. *What was your experience in working with doctors at PHCs? Please share your positive as well as negative experiences Probe:*  - *Interaction with people, Handling the tablet, filling up the information,Motivate people to take treatment,Any other*  1. In your opinion what were the some key issues that prevent people from accessing care and follow up. Probe:  - *What challenges you faced for motivating people to go for treatment?* - *Do you think that conducting camps were effective strategy? If yes. Why??*  1. In your opinion, what were the benefits or problems in using the  - *mhGAP tool? Probe: implementation, Length, content and language of the questionnaire* - *IVR messages ?Probe: implementation, content and language of the message*  1. In your opinion, what are some of the challenges or difficulties that you are encountering with the program? Please give details.   **Suggestions**   1. Please give your suggestions to improve the programme in future? |
| **IDI for community leaders** |
| 1. Name 2. Age 3. Gender 4. Village 5. Give us your opinion about the Smart Mental Health Programme conducted by George Institute, Hyderabad in your community. 6. Can you recall any of the activities *(Probe: ASHAs visit, screening, IVR messages and medical camps).*  - *Did you attend /participated in any of the activities.* - *Did you share the information with your family, friends, colleagues or neighbours? If yes, what was their opinion? If no, what were the reasons?*  1. In your opinion what are the various factors that encouraged and discouraged individuals in seeking treatment?  *Probe: age groups, gender and socio, economic status*   **Screening & Camps**   1. What was your experience when ASHAs visited households for asking the questions/ screening? Please give details  - *Was it useful? If yes, how? If no, please elaborate what was not useful? What can be improved?*  1. Do you think ASHAs are the best person to ask these questions? If yes, why ?If no, please suggest who will be the best person to ask these questions 2. In your opinion, whether people visit the doctor at PHC for treatment? Please share your experience?  - *Was it useful? If yes, how? If no, please elaborate what was not useful? What can be improved?*  1. What was the support people got from the ASHAs and George Institute staff to visit the PHC?  - *Was it useful or not?? If yes, how? If no, please elaborate what was not useful? What can be improved?*  1. In your opinion what are the reasons for follow up patients visiting or not visiting the PHC for continuing treatment? 2. Are you aware of medical camps organized by George Institute in your community??If yes, Please share your experience?  - *Were these camps easy to access?* - *Were doctors easy to approach and consult?* - *Would you like to receive such services in future also? If yes, why?*   **Suggestions**   1. Do you think this programme has any effect- positive/negative on the community? Do you think it must be continued, modified, or discontinued? State your reasons. 2. Please give your suggestions to improve the programme in future? |
| **IDI for doctors** |
| 1. Name 2. Age   **Training**   1. Please tell us about your experience with Smart Mental Health Programme conducted by George Institute Hyderabad. *Probe*  - *Role in programme,* - *interaction with project staff, ASHAs, and community members*  1. How was your experience with training programme conducted by George Institute? *Probe:*  - *How useful or not useful was it?* - *How it helped you professionally?* - *How it can be improved?*  1. What were the aspects and issues that you felt needed more discussion? 2. Do you have any suggestions for future training programme**?**   **App/IVR**   1. How was your experience in using the app/tablet provided by the George as part of project activity? *Probe:*  - *Was training helpful in handling apps and tabs, likes and discomforts?* - *What level of support did you receive from GI staff to handle app/tablet and how?* - *What were the positive and negatives?* - *How do we improve this support?*  1. Did you receive IVR messages? If yes, how was your experience in receiving the IVR messages*? Probe*  - *What were the positive and negative experiences?* - *How do we improve this? If no, than please share the reason for not receiving it.*  1. In your opinion , what were the issues related to the questionnaire ?*Probe: Length, content and language of the questionnaire*   **Implementation**   1. In your opinion what are the reasons for follow up patients revisiting or not visiting the PHC for continuing treatment? 2. What was the level of support you received from GI staff? Probe  - *Do you think this support was useful* - *How can it be improved*  1. In your opinion what were the some key issues that prevent people from accessing care and follow up. Probe:  - *Do you think that conducting camps were effective strategy*   **Suggestions**   1. Please give your suggestions to improve the programme in future interventions? |
